# Assessing the utilization of cancer medicines in Rwanda: an analysis of treatment patterns

**DOI:** 10.3332/ecancer.2023.1631

**Published:** 2023-11-17

**Authors:** Fidel Rubagumya, Brooke Wilson, Cyprien Shyirambere, Achille Manirakiza, Pacifique Mugenzi, Mary Chamberlin, Wilma M Hopman, Christopher Booth

**Affiliations:** 1 Department of Oncology, Rwanda Military Hospital, KK 739 Street, Kicukiro, PO BOX 4016, Kigali, Rwanda; 2 Research for Development (RD) Rwanda, Kigali, Rwanda; 3 Division of Cancer Care and Epidemiology, Queen’s University Cancer Research Institute, Kingston, ON K7L 3N6, Canada; 4 Department of Oncology, Queen’s University, Kingston, ON K7L 3N6, Canada; 5 School of Public Health, University of New South Wales, Sydney, NSW 2052, Australia; 6 Butaro Cancer Center of Excellence, Burera, Rwanda; 7 Oncology Unit, Department of Medicine, King Faisal Hospital, PO BOX 2534, Kigali, Rwanda; 8 Dartmouth Cancer Center, Lebanon, NH 03755, USA; 9 Kingston Health Sciences, Kingston, ON, k7l 2v7, Canada

**Keywords:** cancer, LMICs, Rwanda, chemotherapy, utilisation

## Abstract

**Introduction:**

Cancer is a growing public health concern in Africa, especially in low- and middle-income countries (LMICs) like Rwanda. Increased cancer incidences translate into increased utilisation of cancer medicine. Access to affordable cancer medicines in Rwanda is a pressing issue as the National Health Insurance plan does not provide coverage for cancer medicines. In this study, we investigated the utilisation patterns of cancer medicines in Rwanda.

**Methods:**

This retrospective cross-sectional study was conducted at all referral hospitals (*n* = 3) capable of delivering chemotherapy in Rwanda. The data collection was over a period of 6 months, during which a team of trained research assistants reviewed a convenience sample of selected patient charts. Both paper charts and electronic medical records were used to collect patients' data, including cancer type, stage, treatment setting, type of drugs or regimen used and completed cycles. Data were analysed using descriptive statistics.

**Results:**

A total of 630 patients received chemotherapy during the study period and were included. Seventy-seven percent (*n* = 486) were female and mean age was 51 (SD ± 13). Among all patients receiving chemotherapy, 43% (*n* = 270) had breast cancer, 22% (*n* = 140) had cervical cancer and 19% (*n* = 121) had colorectal cancer. The majority of patients (71%) had a community-based insurance. Butaro Cancer Centre treated the most patients (48%, *n* = 303). Thirty-six percent (221/630) had stage III cancer. The most common regimens within the cohort were adriamycin, cyclophosphamide and taxane, capecitabine and oxaliplatin (CAPOX), paclitaxel + carboplatin and a single agent cisplatin given concurrently with radiotherapy. The proportion of chemotherapy that was given in the curative and palliative setting was 72% and 28% respectively.

**Conclusion:**

Access to affordable cancer medicines remains a challenge in Rwanda. The study's findings provide valuable information on the utilisation patterns of cancer medicines in Rwanda, which can be used to guide policy decisions and improve cancer care in the country.

## Introduction

Cancer is now recognised as a major public health concern in Africa [1]. As populations expand, age and adopt lifestyles that increase cancer risks, the number of cancer diagnoses and deaths is predicted to climb dramatically, especially in low- and middle-income countries (LMICs) such as Rwanda [2]. By 2040, it is anticipated that there will be approximately 30 million new cancer diagnoses and over 16 million yearly cancer-related deaths in LMICs [3]. These new cases will translate into 15 million cases needing chemotherapy, a relative increase of 53% [4]. In 2020, GLOBOCAN estimates suggested Rwanda had a total of 8,835 cancer cases and 6,044 deaths [5]. If all of these patients were treated with high-income resources, chemotherapy utilisation rate would be 57%, and at least 5,000 patients per year would have an indication for chemotherapy [4]. This number may be even higher after adjusting for the advanced stage distributions seen in Rwanda.

Rwanda has witnessed considerable advances in cancer care during the past 20 years. With Butaro Cancer Centre of Excellence (BCCOE), King Faisal Hospital (KFH) and Rwanda Military Hospital (RMH) all providing chemotherapies and other cancer-related services, and RMH providing radiotherapy [6], cancer referrals abroad have reduced. The majority of other tertiary institutions in Rwanda provide surgical and diagnostic services, including pathology and imaging [6, 7]. Despite the availability of these treatments and the fact that more than 80% of the population is covered by community-based health insurance (CBHI) and 15% by private insurance, not every cancer patient in Rwanda has access to affordable cancer medicines [6, 8]. This is mainly because CBHI does not cover cancer medicines.

Access to cancer medicines (availability and affordability) is one of the most pressing issues in emerging cancer systems such as Rwanda [8]. Financial toxicity due to the high costs of cancer medicines is particularly challenging in LMICs [9]; hence, the need for rational utilisation of cancer treatment in low-resource settings. Rational use of cancer drugs includes using them for the correct indication, at the right dosage, and at the lowest cost [10]. Drug utilisation is an essential aspect of cancer care and a critical determinant of treatment outcomes [11]. In LMICs, drug utilisation is often suboptimal, leading to decreased treatment efficacy and increased morbidity and mortality [12]. This can be attributed to a number of factors, including lack of access to essential medicines, limited availability of cancer treatment services, high treatment costs, limited healthcare infrastructure, minimal dose intensity of therapy, low completion rates of scheduled chemotherapy cycles, and a lack of trained personnel [12]. One way to ensure the rational use of cancer medicine is drug utilisation evaluation, which is a systematic evaluation of drug usage that will help ration the use of medication at the individual patient level, particularly for medical, social and economic reasons [10].

In this study, we analyse of the utilisation patterns of cancer medicines in Rwanda with the aim of understanding which chemotherapy regimens were commonly used in Rwanda and in which settings (i.e., neoadjuvant, adjuvant and palliative). This work will guide future policy decisions and ultimately enhance the quality of cancer care.

## Methods

### Study design and sites

Rwanda is a landlocked low-income country in East Africa. The national population is approximately 13.2 million, with Kigali the capital city having 1.7 million (13%) [13]. The current life expectancy is 69 years [13]. More than 90% of patients are covered by CBHI which covers some cancer services [6]. However, this insurance does not cover the costs of cancer drugs. Currently, there are eight referral/tertiary hospitals, four of them being University Teaching Hospitals.

This is a retrospective study conducted at the only three referral hospital with a capacity to treat cancer either by chemotherapy or radiotherapy. These are RMH, KFH and BCCOE. KFH operates under a public-private partnership model while the others are fully public institutions.

RMH offers radiotherapy and chemotherapy and both BCCOE and KFH also offer chemotherapy services. BCCOE is located out of the capital city in the Northern province while RMH and KFH are found in the capital city – Kigali. The data collection was over a period of 6 months from 1 June to 1 December 2022 during which a team of trained research assistants reviewed a convenience sample of patient charts. This study was approved by the Rwanda National Ethics Committee.

### Study population and inclusion criteria

The eligibility criteria were as follows: both female and male patients aged over 18 years with available medical records, diagnosis of breast, cervix, colorectal, Hodgkin's and non-Hodgkin's lymphoma. These specific cancers were selected because the management often includes the use of systemic therapy. Eligible patients were those treated at one of the three sites between January 2018 and December 2021. Children under 18 years and patients whose files had no information about chemotherapy utilisation were excluded

### Data collection

Detailed chart reviews were performed for a convenience sample of patients treated with chemotherapy during 2018–2021 at three centres in Rwanda. Patients’ data were retrieved from both paper charts and electronic medical records. The extracted information included: basic demographic data (age, gender and residence), type of insurance patient had, cancer type/disease site, stage, treatment setting and intent (whether neoadjuvant, adjuvant, palliative or definitive), type of drugs or regimen used. The data capture form was developed after consulting local oncologists and literature on cancer medicine utilisation in other settings. We conducted a pilot test with 15 sample cases of all cancer types of interest. Regular quality checks were conducted by the study principle investigator to ensure data quality and to identify any errors or inconsistencies.

### Data analysis

Data were collected in Qualtrics and exported into IBM SPSS (version 27.0 for Windows, Armonk, NY, USA, 2022) for statistical analysis. Data were analysed using descriptive statistics, including frequencies and percentages for categorical data; means (standard deviations) or medians (quartiles) for continuous data; and cross-tabulations for assessing distributions within pairs of variables.

## Results

### Patient demographics

A total of 630 patients were reviewed and included in this study; characteristics of the study cohort are shown in Table 1. These patients represent approximately 19% (630/3350) of all patients treated with chemotherapy at the three centres (RMH = 857; KFH = 255; BCCOE = 2154) during 2018–2021 according to hospital registry data. Seventy-seven percent (*n* = 486) were female and the most common cancers were 43% (*n* = 270) breast, 19% (*n* = 121) colorectal, 22% (*n* = 140) cervical, 5% (*n* = 34) Hodgkin’s lymphoma and 9% (*n* = 59) non-Hodgkin’s lymphoma, with 1% (*n* = 6) not reporting disease site. The mean age of the study cohort per disease type was 50 years for breast cancer, 55 years for colorectal cancer, 55 years for cervical cancer, 39 years for Hodgkin’s lymphoma and 49 years for non-Hodgkin’s lymphoma. One-quarter of patients (27%, *n* = 169) resided in Kigali. The majority of patients (71%) used the CBHI. Among the study cohort, Butaro Cancer Centre treated the most patients (48%, *n* = 303), with KFH treating the fewest (12%, *n* = 73). However, 78% (*n* = 109) of cervical cancer patients were treated at RMH, the only facility with radiotherapy services (Table 2).

### Stage distribution

More than half of patients had advanced disease, regardless of tumour type. Approximately 36% (221/630) stage III and 24% (150/630) stage IV disease. Stratifying by disease type (Table 3), the proportion of patients with breast cancer receiving chemotherapy with stage III/IV disease was 22%, compared to 14% stages III/IV disease for colorectal cancer, 16% for cervical cancer, 4% for Non-Hodgkin's Lymphoma (NHL) and only 3% for Hodgkin's Lymphoma (HL).

### Chemotherapy utilisation

Among 630 patients that received chemotherapy, drug details were available for 94% (*n* = 594). A complete description of the chemotherapy drugs used, stratified by treatment intent and tumour type, are provided in Table 4. The most common regimens within the cohort were adriamycin, cyclophosphamide and taxane (AC-T) for breast cancer, capecitabine and oxaliplatin (CAPOX) for colorectal cancer, paclitaxel + carboplatin for cervical cancer, and a single agent concurrent cisplatin for cervical cancer. The proportion of chemotherapy that was given in the curative and palliative setting was 73% (*n* = 433) and 27% (*n* = 161), respectively. Temporalising chemotherapy was given to patients with cervical waiting for definitive chemoradiotherapy. The most commonly prescribed regimen for breast cancer was AC-T (*n* = 192/264) with 41% (*n* = 78/192) being prescribed in the neoadjuvant setting, 50% (*n* = 96/192) adjuvant and 9% (*n* = 18/192) in the palliative settings. For colorectal cancer, CAPOX regimen was most commonly prescribed (49%, *n* = 54/110) with 9% (*n* = 5/54) as neoadjuvant, 35% (*n* = 19/54) as adjuvant and 56% (*n* = 30/54) as palliative. For NHL, cyclophosphamide, doxorubicin, vincristine and prednisolone (CHOP) was most commonly used (79%, *n* = 44/56) while doxorubicin, bleomycin, vinblastine and dacarbazine (ABVD) was commonly used for HL.

## Discussion

In this report, we describe the real-world delivery of chemotherapy across Rwanda. Several important findings emerged. First, breast, cervix and colorectal cancer accounted for the highest proportion of chemotherapy utilisation among the included cancer types. Second, the most common regimens used were ACT for breast cancer and CAPOX for colorectal cancer. Third, the BCCOE receives the greatest number of patients among the three cancer centres in Rwanda. Fourth, most patients present with very advanced disease. Finally, most chemotherapy delivery is given in the context of curative intent therapy.

It is useful to consider our results in the context of other studies. In comparison to our study (33% of neoadjuvant chemotherapy (NACT) for breast cancer), an analysis of 388 patients with cancer seen from 2005 to 2014 at the National Radiotherapy Oncology and Nuclear Medicine Centre in Ghana showed that 41% of patients with breast cancer received NACT [14]. Our findings are contrary to a study evaluating cancer utilisation in Saudi Arabia that found most chemotherapy to be utilised in neoadjuvant settings [12]. Additionally, our findings are different from those of Onitilo *et al* [15], who conducted a study on the use of NACT in breast cancer patients treated in four institutions across the United States from January 2003 to December 2008 and discovered that only 4% (111/2907) patients were given NACT. Furthermore, our findings are different from data from Butaro Cancer Center where they found that rates of exclusive neoadjuvant utilisation in breast cancer both early and locally advanced was 20% (29/145) while exclusive adjuvant utilisation was 21% in the same cohort [16]. The noted difference in chemotherapy utilisation in these two cohorts can be explained by the fact that O’Neil *et al* [16] reviewed data within year of Butaro Cancer Center establishment where most patients who presented then were very advanced and metastatic disease whose management was only palliative as compared to a cohort of 2018–2021. Our findings on NACT use are supported by stage distribution data, where 36% of our population had stage III disease at diagnosis. Hence the need to use NACT to improve surgical resection rates. This implies that a large proportion of patients get appropriate care. Different from a study done in Saudi Arabia looking at the drug utilisation and expenditure of anticancer drugs for breast cancer, 80% of patients utilised fluorouracil, epirubicin and cyclophosphamide (FEC) [12], in our study ACT was most commonly utilised, which is consistent with current national and international cancer guidelines [17]. In contrast, regional variation within Africa exists, and a recent study from Ghana found 72% used doxorubicin, cyclophosphamide and fluorouracil, while ACT was only used in 23% [14]. This study also found most patients had advanced stage presentation, with approximately 73% of breast cancer patients having node positive disease [14].

We found that patients are equally distributed across all provinces. However, it is worth noting that a majority use CBHI for care, which unfortunately does not cover cancer drugs. This may explain the high number of patients being treated at Butaro Cancer Center, which provides cancer drugs free of charge through private philanthropic funding from Partners in Health. Despite drugs being given free of charge, frequent travel to BCCOE has both financial and clinical implications. It is known that distance from cancer centres can lead to treatment abandonment [18, 19]. Transportation needs, as well as days spent not working, can be major sources of financial burden [20, 21]. This calls for decentralised cancer care, which can only be possible if cancer drugs are covered by National Health Insurance, which would allow provincial and other referral hospitals in Rwanda to administer chemotherapy. However, this would require proper planning from a workforce and infrastructure perspective.

With the high proportion of late stage diagnoses, the use of NACT in colorectal and breast could be improved given that in some circumstances it might allow organ preservation. It could also minimise side effects caused by additional radiotherapy for some breast cancer patients. If used correctly, Neo-adjuvant chemotherapy (NAC) and endocrine therapy can also reduce the need for radiotherapy, allowing those who require it to use it [22]. This calls for a policy to enforce adherence to national cancer treatment guidelines.

The majority of chemotherapy administration in our cohort is utilised in the context of curative intent therapy. This observation may be attributed to the pragmatic approach adopted by the BCCOE [23], which has a high number of patients within this cohort. The limited availability of resources necessitates the prioritisation of patients with curative cases and a selective approach towards treating palliative cases. BCCOE made this decision at its inception and guidelines were created on what type of cancer can be treated and what regimens can be utilised [23]. As the capacity increases, different indications are added to these guidelines.

Our findings should be interpreted with methodological limitations in mind, particularly those inherent in retrospective studies. First, it should be noted that due to pragmatic limitations in study staffing (leading to the randomly selected convenience sampling approach), this study only includes 19% of patients who received treatment from 2018 to 2021. Our results are based on a convenience sample of patients charts and therefore may not be generalisable to all chemotherapy given in Rwanda. Moreover, our selection criteria focused on patients with breast, cervix, colorectal cancer and lymphoma; as a result, little is known about chemotherapy treatment patterns for patients with other primary tumour types. Second, there were missing data, for example, 24% of patients did not have stage mentioned in their charts. Third, this paper does not report on the treatment outcomes to see the impact of stages, and the role of treatment settings (neoadjuvant versus adjuvant). Fourth, two of the four study years included the COVID-19 pandemic period which very likely was associated with temporal differences in chemotherapy use. Finally, in retrospect, we could have added rates of completion or abandonment and dose intensity. Despite these limitations, this is the first paper to report on the use of chemotherapy in Rwanda, covering the five cancers that heavily rely on chemotherapy. Additionally, it covers all the centres that provide cancer chemotherapy in Rwanda. Future prospective studies are needed to expand the cohort size and will ideally use mixed qualitative/quantitative methods to expand on the insights that can be gained from patients. Further studies are also needed to evaluate the rational use of cancer drugs and the financial and clinical implications of expanded drug access through public insurance schemes.

Our findings have policy implications. First, the high utilisation of chemotherapy for breast, cervix and colorectal cancer underscores the importance of prioritising prevention and early detection efforts to improve patient outcomes. Second, the prominence of the BCCOE despite the location highlight the need to include cancer drugs on the National Health Insurance – CBHI. Third, a high proportion of advanced stages calls for urgency of implementing strategies that promote screening and early detection of cancer to improve rates of late presentation.

## Conclusion

Given the increasing number of cancer cases in Rwanda, and limited resources available, it is critical that rational use of cancer chemotherapy be incorporated into the national cancer control plan. Using cancer medications with the appropriate indication and regimen at the lowest possible cost is an example of rational drug use. It should be noted that irrational use includes ineffective treatment, unnecessary prescription of drugs, severe adverse effects and financial burden. One way to improve utilisation is to use treatment algorithms, make all essential drugs available on the national formulary and increase training. This study provides a snapshot of which cancer medicines are most commonly used on the front lines of clinical care in Rwanda. This data can be used to plan service expansion and to estimate the costs and demands of providing a comprehensive package of publicly funded chemotherapy drugs for patients with common malignancies treated with chemotherapy in Rwanda.

## Conflicts of interest

The authors declared no conflicts of interest.

## Funding

This study was not funded.

## Figures and Tables

**Table 1.** Characteristics of 630 patients treated with chemotherapy at three centres in Rwanda during 2018–2021.

	% (*N*)
Gender distribution	
Male	77 (486)
Female	22 (139)
Missing	1 (5)
Cancer site	
Breast	43 (270)
Colorectal	19 (121)
Cervical	22 (140)
Hodgkin's lymphoma	5 (34)
Non-Hodgkin's lymphoma	9 (59)
Missing	1 (6)
Province of residence	
East	17 (110)
West	18 (116)
South	19 (122)
North	18 (111)
Kigali	27 (169)
Missing	0 (2)
Type of insurance schemes	
Mituelle	71 (447)
RSSB	9 (57)
MMI	7 (43)
FARG	5 (28)
Other^a^	8 (51)
Missing	1 (4)

RSSB: Rwanda Social Security Board, MMI: Military Medical Insurance, FARG: Fonds d'Assistance aux Réscapés du Génocide (Genocide Survivors Assistance Fund)

a Britam: British-American Asset Managers Kenya Limited, UAP: Union des Assurances de Paris, SANLAM: Suid-Afrikaanse Nasionale Lewens Assuransie Maatskappij, Rwanda Bar Association (BAR-Association)

**Table 2.** Cancer diagnoses among patients treated with chemotherapy during 2018–2021 the three referral hospitals (N-619).

Distribution of cases among cancer centres % (*N*)			
	RMH	KFH	Butaro
Breast cancer	22 (54)	42 (30)	61 (185)
Colorectal	22 (54)	45 (32)	11 (34)
Cervical	45 (109)	1 (1)	10 (29)
Hodgkin's lymphoma	6 (14)	8 (6)	4 (13)
Non-Hodgkin’s lymphoma	5 (13)	4 (3)	14 (42)
Total	244	72	49 (303)

Five were missing hospital and 6 were missing disease site

**Table 3.** Stage distribution per disease type (*N* = 616).

	Breast % (N)	Colorectal % (N)	Cervix % (N)	HL % (N)	NHL % (N)
Stage I	4 (10)	6 (7)	3 (4)	5 (2)	10 (6)
Stage II	6 (17)	8 (10)	26 (36)	18 (6)	3 (2)
Stage III	31 (83)	31 (37)	57 (78)	29 (10)	21 (12)
Stage IV	22 (58)	43 (51)	13 (18)	24 (8)	24 (14)
Unstaged	37 (100)	12 (14)	0 (0)	24 (8)	42 (25)
Total	268	119	136	34	59

Stage was missing for nine and disease site was missing for six; one was missing data for both. Unstaged are patients who were not staged prior to starting chemotherapy

**Table 4.** Type of chemotherapy regimen per treatment intent (*N* = 594).

Disease	Regimen/drug	Neoadjuvant	Adjuvant	Palliative (first line)	Definitive	Temporalising	Total
Breast							
	ACT-T	78	96	18	0	0	192
	Paclitaxel + carboplatin	2	1	7	0	0	10
	Others	2	5	55	0	0	62
	Total	82	102	80	0	0	264
Colorectal							
	FOLFOX	8	22	12	0	0	42
	CAPOX	5	19	30	0	0	54
	Others	-	4	10	0	0	14
	Total	13	45	52	0	0	110
Cervix							
	Cisplatin + radiation	4	-	1	58	-	63
	Paclitaxel + carboplatin	9	-	17	2	38	66
	Others	-	-	1	2	-	3
	Total	13	-	19	62	38	132
Hodgkin's lymphoma							
	ABVD	-	-	-	14	0	14
	Others	-	-	-	18	-	18
	Total	-	-	-	32	0	32
Non-Hodgkin's lymphoma							
	CHOP	1	2	2	39	0	44
	CHOP-R	1	0	8	0	0	9
	Others	0	0	0	3	0	3
	Total	2	2	10	42	0	56
Total							594

Disease site was missing for 6 and treatment intent was missing for 31 (one was missing data for both)
